# Genomic Predictors of Brisk Walking Are Associated with Elite Sprinter Status

**DOI:** 10.3390/genes13101710

**Published:** 2022-09-23

**Authors:** João Paulo L. F. Guilherme, Ekaterina A. Semenova, Andrey K. Larin, Rinat A. Yusupov, Edward V. Generozov, Ildus I. Ahmetov

**Affiliations:** 1 Laboratory of Applied Nutrition and Metabolism, School of Physical Education and Sport, University of São Paulo, São Paulo 05508-030, Brazil; 2 Laboratory of Biochemistry and Molecular Biology of Exercise, School of Physical Education and Sport, University of São Paulo, São Paulo 05508-030, Brazil; 3 Department of Molecular Biology and Genetics, Federal Research and Clinical Center of Physical-Chemical Medicine of Federal Medical Biological Agency, 119435 Moscow, Russia; 4 Research Institute of Physical Culture and Sport, Volga Region State University of Physical Culture, Sport and Tourism, 420138 Kazan, Russia; 5 Department of Physical Culture and Sport, Kazan National Research Technical University Named after A.N. Tupolev-KAI, 420111 Kazan, Russia; 6 Department of Physical Education, Plekhanov Russian University of Economics, 115093 Moscow, Russia; 7 Laboratory of Molecular Genetics, Central Research Laboratory, Kazan State Medical University, 420012 Kazan, Russia; 8 Sports Genetics Laboratory, St. Petersburg Research Institute of Physical Culture, 191040 St. Petersburg, Russia; 9 Research Institute for Sport and Exercise Sciences, Liverpool John Moores University, Liverpool L3 5AF, UK

**Keywords:** DNA, genotype, athletes, athletic status, strength, height, muscle mass, sprint performance, muscle fiber composition, walking pace

## Abstract

Brisk walkers are physically more active, taller, have reduced body fat and greater physical fitness and muscle strength. The aim of our study was to determine whether genetic variants associated with increased walking pace were overrepresented in elite sprinters compared to controls. A total of 70 single-nucleotide polymorphisms (SNPs) previously identified in a genome-wide association study (GWAS) of self-reported walking pace in 450,967 European individuals were explored in relation to sprinter status. Genotyping of 137 Russian elite sprinters and 126 controls was performed using microarray technology. Favorable (i.e., high-speed-walking) alleles of 15 SNPs (*FHL2* rs55680124 C, *SLC39A8* rs13107325 C, *E2F3* rs4134943 T, *ZNF568* rs1667369 A, *GDF5* rs143384 G, *PPARG* rs2920503 T, *AUTS2* rs10452738 A, *IGSF3* rs699785 A, *CCT3* rs11548200 T, *CRTAC1* rs2439823 A, *ADAM15* rs11264302 G, *C6orf106* rs205262 A, *AKAP6* rs12883788 C, *CRTC1* rs11881338 A, *NRXN3* rs8011870 G) were identified as having positive associations with sprinter status (*p* < 0.05), of which *IGSF3* rs699785 survived correction for multiple testing (*p* = 0.00004) and was linked (*p* = 0.042) with increased proportions of fast-twitch muscle fibers of m. vastus lateralis in physically active men (*n* = 67). Polygenic analysis revealed that individuals with ≥18 favorable alleles of the 15 SNPs have an increased odds ratio of being an elite sprinter when compared to those with ≤17 alleles (OR: 7.89; *p* < 0.0001). Using UK Biobank data, we also established the association of 14 favorable alleles with low BMI and fat percentage, 8 alleles with increased handgrip strength, and 7 alleles with increased height and fat-free mass. In conclusion, we have identified 15 new genetic markers associated with sprinter status.

## 1. Introduction

Walking is a simple form of physical exercise with major impacts on human biology. Increasing walking pace improves certain aspects of human physiology [1]. Compared to individuals who walk slowly, brisk walkers are physically more active, taller, have reduced waist circumference and body fat, and greater cardiorespiratory fitness and muscle strength, which implies improvement in a wide range of important health conditions [1,2,3]. Walking pace can be modified by lifestyle behaviours, but it is also an inherited trait, mediated by genetic variants [4]. Inherited genetic variants may contribute to an individual’s predisposition to spontaneously walk faster.

A recent genome-wide association study (GWAS) of self-reported walking pace using data from 450,967 individuals of European ancestry—participants self-reported their walking pace as “slow” (<3 mph), “steady/average” (3–4 mph) or “brisk” (>4 mph)—identified 70 independent genetic loci (Supplementary Table S1) associated with self-reported walking pace at a genome-wide significance level [4]. The genetic findings for self-reported walking pace overlapped highly with several other traits, including cognitive outcomes, adiposity, cardiometabolic factors and muscle strength [4]. Of particular interest, a strong genetic correlation was observed between self-reported walking pace and handgrip strength, even after adjusting for body mass index (BMI), suggesting a shared genetic basis for these traits [4]. There is a close relationship between muscle strength, height and walking speed [3,5,6,7], and all of them are key intermediate phenotypes for sprinters [8]. Sprint speed is a product of step length and step rate; to sprint fast, first of all, one needs to be able to generate large strides, which requires muscle strength [9]. As such, it seems plausible to suppose that genetic loci associated with walking pace can also predispose an individual to perform better in sprint exercises. However, these genetic components have not yet been evaluated in athletes.

Due to the polygenic and complex nature of sports performance, identifying the genetics of top-level athletes has been more difficult than initially envisaged. A GWAS involves the scanning of several hundred thousand genetic markers across individual genomes to find genetic variations associated with a particular trait [10]. As a consequence, a massive amount of information can be generated by a GWAS, which needs to be replicated in independent studies to validate or reinforce the proposed findings. In recent years, some GWASs were carried out in athletes [11,12,13,14], with some validation of initial findings [12,15,16]. Nevertheless, very robust investigations have been carried out in non-athletic populations with a diverse array of intermediate phenotypes which may also be interesting with respect to the predisposition of individuals to excellence in sport, such as self-reported walking pace. A large number of genetic variants contribute to the genetic architecture of elite athletes and the replication of data generated by a GWAS in non-athletic populations will substantially contribute to the discovery of novel DNA polymorphisms explaining the innate characteristics of athletes.

The present study aimed to explore the association between single-nucleotide polymorphisms (SNPs) related to self-reported walking pace and sprint athlete status. A total of 70 SNPs previously associated with self-reported walking pace at *p* < 1.5 × 10^−8^ [4] were compared between elite sprinters and ethnically matched controls. SNPs were evaluated individually or combined using a genotype score (additive effect of alleles). We explored the hypothesis that high-speed-walking alleles (HSWAs) are overrepresented in elite sprinters and might be associated with intermediate phenotypes, such as handgrip strength, muscle fiber composition, height, fat-free mass, BMI and body fat percentage.

## 2. Materials and Methods

### 2.1. Ethical Approval

The study was approved by the Ethics Committee of the Federal Research and Clinical Center of Physical–Chemical Medicine of the Federal Medical and Biological Agency of Russia (protocol no. 2017/04). Written informed consent was obtained from each participant. The study complied with the Declaration of Helsinki and ethical standards for sport and exercise science research.

### 2.2. Study Participants

A total of 263 Russian subjects participated in a case–control study: 137 athletes (84 men and 53 women; mean age ± SD: 24.7 ± 3.7 years) and 126 controls (80 men and 46 women; mean age ± SD: 43.1 ± 3.7 years). The athletes were all elite sprinters from the following sport disciplines: canoeing 200–500 m (*n* = 8), kayaking 200–500 m (*n* = 21), running 100–400 m (*n* = 27), short track 500–1000 m (*n* = 3), speed skating 500–1000 m (*n* = 31), sprint cycling (*n* = 22) and swimming 50–100 m (*n* = 25). The athletes were Russian national team members (participants and prize winners in international competitions) who had never tested positive for doping. The controls were healthy, unrelated Russians without any competitive sport experience. The muscle biopsy study involved 67 physically active men of Russian origin (mean age ± SD: 32.9 ± 8.9 years; mean height: 180.4 ± 6.1 cm; mean body mass: 77.3 ± 8.4 kg; mean percentage of fast-twitch muscle fibers: 49.4 ± 17.7%; mean percentage of slow-twitch muscle fibers: 54.1 ± 17.8%; cross-sectional area (CSA) of slow-twitch muscle fibers: 5307 ± 1298 μm^2^; CSA of fast-twitch muscle fibers: 5423 ± 1440 μm^2^).

### 2.3. Evaluation of Muscle Fiber Composition by Immunohistochemistry

**Figure 1:**
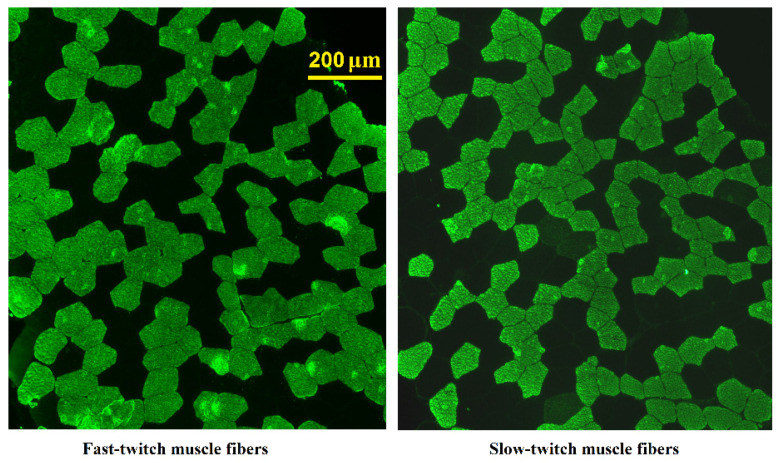
Microphotographs of the labelled muscle sections.

Vastus lateralis samples were obtained from the left leg using the modified Bergström needle procedure with aspiration under local anaesthesia using 2% lidocaine solution. Prior to analysis, samples were frozen in liquid nitrogen and stored at −80 °C. Serial cross-sections (7 μm) were obtained from frozen samples using an ultratom (Leica Microsystems, Wetzlar, Germany). Sections were thaw-mounted on Polysine glass slides, maintained at room temperature (RT) for 15 min and incubated in PBS (3 × 5 min). The sections were then incubated at RT in primary antibodies against slow or fast isoforms of the myosin heavy chains (M8421, 1:5000; M4276; 1:600, respectively; Sigma-Aldrich, Burlington, MA, USA) for 1 h and incubated in PBS (3 × 5 min). Afterwards, the sections were incubated at RT in secondary antibodies conjugated with FITC (F0257; 1:100; Sigma-Aldrich) for 1 h. The antibodies were removed, and the sections washed in PBS (3 × 5 min), placed in mounting media and covered with a cover slip. Images were captured with a fluorescent microscope (Eclipse Ti-U, Nikon, Tokyo, Japan). All analysed images contained 328 ± 12 fibers. The ratio of the number of stained fibers to the total fiber number was calculated. Fibers stained in serial sections with antibodies against slow and fast isoforms were considered hybrid fibers (Figure 1). The cross-sectional areas (CSAs) of fast- and slow-twitch muscle fibers were evaluated using ImageJ software (NIH, Bethesda, MD, USA).

### 2.4. Genotyping

Molecular genetic analysis was performed with DNA samples obtained from leukocytes (venous blood). Four milliliters of venous blood was collected in tubes containing EDTA (Vacuette EDTA tubes, Greiner Bio-One, Kremsmünster, Austria). Blood samples were transported to the laboratory at 4 °C, and DNA was extracted on the same day. DNA extraction and purification were performed using a Technosorb commercial kit (Technoclon, Moscow, Russia), according to the manufacturer’s instructions. Genotyping of 70 SNPs was performed using microarray technology (HumanOmni1-Quad BeadChips, Illumina, San Diego, CA, USA), as previously described [17].

Self-reported walking pace SNPs associated with sprint athlete status were further tested for associations with sports-relevant traits (e.g., height, fat-free (lean) mass and handgrip strength) in the UK Biobank—a prospective population-based study, whose summary statistics are available from https://genetics.opentargets.org/(accessed on 6 September 2022). Data retrieved from the UK Biobank, particularly associations with handgrip strength, height, fat-free mass, BMI, fat percentage and other phenotypes, are shown in Supplementary Table S2.

To assess the combined association of SNPs, we used a mathematical model based on the sum of favorable alleles, termed the total genotype score (TGS), as originally employed [18]. Briefly, based on the phenotype of interest, the homozygous-associated genotype received a score of 2, while the heterozygote received a score of 1, and the alternate homozygous genotype received a score of zero. The scores for each polymorphism were summed to generate the total score, which was transformed into a scale ranging from 0 to 100 by dividing the total score by the maximum possible score and multiplying by 100. Greater values indicate a more favorable polygenic profile.

### 2.5. Statistical Analyses

Statistical analyses were conducted using GraphPad InStat Version 3.05 (GraphPad Software, Inc., San Diego, CA, USA; www.graphpad.com (accessed on 6 September 2022)) software. The chi-square test (χ^2^) was used to test for the presence of the Hardy–Weinberg equilibrium (HWE). Thereafter, the frequencies of genotypes or alleles were compared between sprinters and controls using the χ^2^ or Fisher’s exact test. The most frequent (major) allele was called allele 1, while the less frequent (minor) allele was called allele 2. Thus, for each SNP, three genotypes were observed: 1/1 (major genotype), 1/2 (heterozygous genotype) and 2/2 (minor genotype). Three genetic models were used to compare the genotype distribution: a codominant model (1/1 vs. 1/2 vs. 2/2), a dominant model (1/1 vs. 1/2 + 2/2) and a recessive model (1/1 + 1/2 vs. 2/2). A nominal association was set at *p* < 0.05, and Bonferroni correction for multiple testing was performed when appropriate (i.e., *p* < 0.00018 [0.05/70 SNPs*4 models]). A descriptive analysis of the TGS was performed, and the mean differences between groups were compared using an unpaired *t*-test. Receiver-operating characteristic (ROC) curves were plotted and the areas under the curves (AUCs) were calculated to assess the discriminative power of the TGS. Multiple regression was used to assess the relationships between genotypes and proportions of fast-twitch muscle fibers (adjusted for age). Muscle fiber composition data are presented as means (SDs).

## 3. Results

### 3.1. Case–Control Study

**Figure 2:**
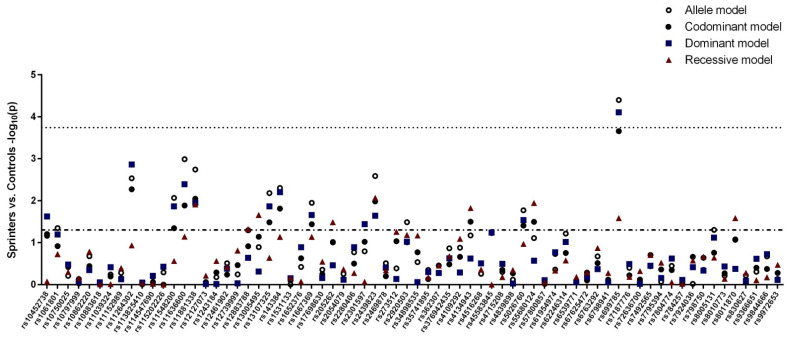
Genetic association tests of single-nucleotide polymorphisms (SNPs) related to self-reported walking pace between elite sprinters and ethnically matched controls. The *y*-axis represents the negative log10-transformed *p*-values of each of the evaluated genetic models, and the *x*-axis indicates the SNPs, according to the reference SNP ID numbers (rs nos.). The dashed line represents the significance threshold for nominal associations (*p* < 0.05), and the dotted line represents the significance threshold adjusted for multiple comparisons (*p* < 0.00018).

A total of 20 SNPs showed a nominal association (*p* < 0.05) with sprinter status in at least one of the evaluated models (allele model, codominant model, dominant model or recessive model), as shown in Figure 2. While five SNPs (rs13005495, rs5026760, rs1061801, rs8005131 and rs2301597) showed an association in the opposite direction to the study hypothesis, as evidenced by the negative difference in the prevalence of the HSWAs between sprinters and controls, 15 SNPs had associations that were in line with the study hypothesis (i.e., HSWAs were overrepresented in athletes).

Table 1 describes the 15 SNPs previously associated with self-reported walking pace that were also associated with sprinter status in our cohort, and the prevalence of the HSWAs in sprinters and controls.

Table 2 shows the genotype distribution in athletes and controls for the 15 SNPs associated with sprinter status. Based on our study cohort, some SNPs (rs11264302, rs699785, rs2920503, rs4134943, rs2439823 and rs143384) showed evidence of a codominant effect, mainly driven by a higher frequency of the minor allele in sprinters (genotype distribution (%): ↑ 2/2; ↑ 1/2; ↓ 1/1), where allele 1 refers to the major allele and allele 2 refers to the minor allele, as shown in Table 2. The SNP rs11881338 also showed a higher frequency in athletes carrying the minor allele. On the other hand, for some SNPs, the association was mainly driven by a higher frequency of athletes homozygous for the major allele (rs11548200, rs55680124, rs13107325, rs10452738 and rs1667369) or athletes carrying the major allele (rs205262, rs12883788 and rs8011870). Of note, the *IGSF3* rs699785 was the most frequently associated SNP and remained significant after correction for multiple comparisons (*p* < 0.00018) in two of the four evaluated models. Due to the departure from HWE, rs11636600 (athletes: χ^2^ = 9.9, *p* = 0.0016), rs362307 (controls: χ^2^ = 5.2, *p* = 0.023) and rs7187776 (athletes: χ^2^ = 4.6, *p* = 0.031) were not included in our association tests based on allele frequency (Table 1) and genotype distribution (Table 2).

### 3.2. Polygenic Analysis

To test the strength of association of the SNPs together, two scoring models based on the sum of HSWAs were explored between the athletes and the controls. In the first scoring model, the TGS was computed using all 70 SNPs previously associated with self-reported walking pace [4], whereas in the second scoring model the TGS was computed using the 15 SNPs associated with brisk walking and sprint athlete status.

Table 3 summarizes the TGS models between athletes and controls. Both TGS models were able to differentiate the sprinter group from the control group—higher scores were found more frequently in the athlete group, and therefore the mean ± SD of the TGS in the sprinters was higher compared to controls, as shown in Table 3. However, the groups and models differed in terms of the score distribution curves. Athletes exhibited a score distribution curve close to symmetry in both models (skewness ≈ 0), while the score distribution curve for controls had the tail skewed to the left (negative skew), mainly in the TGS computed using 15 SNPs, where the mean was less than the mode (i.e., a greater dispersion of low scores). Regarding the shape of the score distribution curve (kurtosis), the TGS computed using 70 SNPs showed a platykurtic (flatter) distribution in both groups (athletes and controls), while the TGS computed using 15 SNPs showed a mesokurtic (normal) distribution in controls and a leptokurtic distribution in athletes. A leptokurtic distribution has a higher peak and taller tails than a normal distribution (i.e., a greater chance of higher scores in athletes).

ROC curve analysis confirmed that both TGS models were able to discriminate sprinters from controls; however, the area under the ROC curve of the TGS computed using 15 SNPs (AUC [95% CI]: 0.781 [0.725–0.837]) suggests a better accuracy compared to the TGS computed using 70 SNPs (AUC [95% CI]: 0.673 [0.608–0.738]) (Table 3). When the TGS was computed using 15 SNPs, the control group had scores between 23.3 and 73.3 (7 to 22 favorable alleles) and the athlete group had scores between 40.0 and 86.7 (12 to 26 favorable alleles). Based on this score distribution, it was found that 76.6% of the sprinters had scores ≥ 60.0 (18 favorable alleles) compared to only 29.4% of the controls. Therefore, those with a score ≥ 60.0 had an increased odds ratio (OR) of being a sprinter when compared to those with a score ≤ 56.7 (17 favorable alleles) (OR: 7.89; *p* < 0.0001). As the scoring range for the TGS computed using 70 SNPs was smaller, the best cut-off point was 52.1 (73 favorable alleles), where it was found that 71.5% of the sprinters had scores ≥ 52.1 compared to 44.4% of the controls (OR: 3.14; *p* < 0.0001).

### 3.3. Muscle Biopsy Study

Of the 15 SNPs associated with fast walking and sprint athlete status, only IGSF3 rs699785 has been linked (*p* = 0.042, adjusted for age) with muscle fiber composition of m. vastus lateralis in physically active men (*n* = 67) (Supplementary Table S4). More specifically, the high-speed-walking allele (IGSF3 rs699785 A) has been associated with an increased proportion of fast-twitch muscle fibers.

### 3.4. Bioinformatical Analysis of 15 SNPs

Using UK Biobank data (https://genetics.opentargets.org (accessed on 6 September 2022)), we also established the association of 14 (out of 15) favorable alleles (rs55680124 C, rs13107325 C, rs4134943 T, rs1667369 A, rs143384 G, rs2920503 T, rs10452738 A, rs699785 A, rs11548200 T, rs2439823 A, rs11264302 G, rs205262 A, rs12883788 C, rs11881338 A) with low BMI and fat percentage, 8 favorable alleles (rs55680124 C, rs13107325 C, rs4134943 T, rs1667369 A, rs143384 G, rs2920503 T, rs10452738 A, rs2439823 A) with increased handgrip strength, 7 favorable alleles (rs55680124 C, rs13107325 C, rs4134943 T, rs1667369 A, rs143384 G, rs10452738 A, rs699785 A) with increased height, and 7 favorable alleles (rs55680124 C, rs13107325 C, rs4134943 T, rs1667369 A, rs143384 G, rs2920503 T, rs11548200 T) with increased fat-free mass (Supplementary Table S2). In addition, four favorable alleles (rs55680124 C, rs13107325 C, rs1667369 A, rs699785 A) were found to be protective against falls in the last year in the UK Biobank cohort (Supplementary Table S2). On the other hand, we found several opposite associations, where favorable alleles were associated with decreased fat-free mass (five SNPs), decreased height (one SNP) and decreased handgrip strength (one SNP) (Supplementary Table S2).

## 4. Discussion

The present study aimed to identify the shared genetic background between self-reported walking pace and sprinter status. Since walking speed (e.g., step frequency) has a high correlation with sprint performance variables [8], it appears reasonable to assume that HSWAs can be part of a favorable genetic architecture of elite sprinters and therefore critical for superior sprint performance achievement. Indeed, we identified that 20 out of 70 candidate SNPs were more frequent in elite sprinters compared to ethnically matched controls. Of these, 15 SNPs had the same direction of association between self-reported brisk walking pace and sprint athlete status—HSWAs were overrepresented in sprinters. The most significant SNP (rs699785) was located in the *IGSF3* gene and its favorable allele (i.e., the A allele) was also associated with an increased proportion of fast-twitch muscle fibers (beneficial to sprinting), increased height and decreased BMI.

The SNPs overrepresented in the sprinter cohort are located in or near genes that have multiple functions, including cell adhesion (*ADAM15*), gene transcription regulation (*ZNF568*), growth and development (*GDF5*, *C6orf106*, *CRTAC1*, *E2F3*, *FHL2*), intracellular transport (*SLC39A8*), metabolism (*CRTC1*, *AKAP6*, *PPARG*) and neurodevelopment (*AUTS2*, *CCT3*, *IGSF3*, *NRXN3*). More details of gene functions are presented in Supplementary Table S3.

The biological relevance of most of these 15 genes to human physical performance is not yet known, but their effect on sprinting ability may be mediated through their association with an increased proportion of fast-twitch muscle fibers (*IGSF3*), increased height (*FHL2, SLC39A8, E2F3, ZNF568, GDF5, AUTS2, IGSF3*), increased handgrip strength (*FHL2, SLC39A8, E2F3, ZNF568, GDF5, PPARG, AUTS2, CRTAC1),* increased fat-free mass (*FHL2, SLC39A8, E2F3, ZNF568, GDF5, PPARG, CCT3*) and decreased fat percentage/BMI (*FHL2, SLC39A8, E2F3, ZNF568, GDF5, PPARG, AUTS2, IGSF3, CCT3, CRTAC1, ADAM15, C6orf106, AKAP6, CRTC1*).

*CRTC1* gene, which regulates cAMP response element-binding protein (CREB), is required for dendrite growth in response to neuronal activity [19], and the *CCT3* gene, which encodes a chaperonin complex subunit, is required for neuronal branching, synaptic growth and neuromuscular junction development [20]. In two previous studies, we found that another gene involved in brain and neuromuscular junction development (*CPNE5*) also has an SNP associated with an increased proportion of fast-twitch muscle fibers and sprint performance [13,15].

Within skeletal muscle, the *CRTC1* gene, also known as transducer of regulated CREB activity 1, activates genes involved in the stimulation of mitochondrial biogenesis, nutrient uptake and metabolism, stimulation of hypertrophy, and muscle repair [21]. The *E2F3* gene, which plays a critical role in the cell cycle and cell proliferation, has a pivotal role in muscle and bone development [22]. Partial deletion of the *E2F3* gene resulted in reduced lean mass and poor grip-strength ability [22]. Interestingly, the *E2F3* gene was up-regulated 5 h after resistance exercise for muscle hypertrophy in resistance-trained male subjects [23], as well as up-regulated 2 h 20 min after three all-out cycle sprints of 30 s duration in healthy men and women [24]. *AUTS2* and *ADAM15* were also up-regulated after resistance or sprint exercise, respectively [23,24].

SNPs associated with self-reported walking pace were shown to have genetic correlations with other complex traits, such as cardiometabolic traits, respiratory traits, cognition and, of particular interest to our study, anthropometric traits and muscular strength [4]. Brisk walkers appear to be genetically prone to being thin and strong, which can be advantageous for sprint performance. Of the 70 SNPs previously associated with self-reported walking pace, 34 were nominally associated with handgrip strength in the UK Biobank cohort [25], of which 8 were also associated with sprinter status in our study, including SNPs located in the following genes: *AUTS2*, *CRTAC1*, *E2F3*, *FHL2*, *GDF5*, *PPARG*, *SLC39A8* and *ZNF568*. *SLC39A8* rs13107325 and *CRTAC1* rs2439823 (both in high linkage disequilibrium (LD) with *SLC39A8* rs13135092 and *CRTAC1* rs563296, respectively) were also overrepresented in an independent cohort of strength athletes composed of weightlifters and powerlifters [26]. Interestingly, regarding *SLC39A8*, which encodes a membrane transporter responsible for manganese uptake into the brain, rs13107325-C was one of the strongest self-reported walking-pace signals [4]. While rs13107325-T was identified as a shared allele between obesity aetiology and brain function (potentially implying a dysregulation of the dopamine motive system that contributes to addiction and obesity) [27], rs13107325-C was associated with brisk walking [4], handgrip strength [25], and strength [26] or sprint (the present study) athlete status.

*C6orf106* rs205262 and *GDF5* rs143384 were also previously associated with complex traits relevant to sprint performance. In a population-based prospective health survey, *C6orf106* rs205262-G was associated with long-term change in BMI (β = 0.13 kg∙m^−2^ per effect allele per 10-year follow-up) [28]. In another study, rs205262-G (conditioned on *SNRPC* rs75398113) was less frequent in thin compared to obese individuals [29], which is in line with our findings of reduced rs205262-G frequency in sprinters (supposedly thin individuals). Moreover, *C6orf106* rs205262-G was also strongly associated with leg pain upon walking in 118,905 UK Biobank participants [30], which suggests that carriers of the rs205262-A may experience less discomfort when walking faster.

The *GDF5* gene encodes an extracellular signaling molecule that participates in the development, maintenance and repair of bone, cartilage, and other tissues of the synovial joint [31]. There is also evidence from animal studies that *GDF5* regulates satellite cell proliferation and differentiation [32], which could be important after an intense sprint or strength training session. In addition to the brisk walking, fat-free mass and handgrip strength traits, *GDF5* rs143384-G was also strongly associated with sitting or standing height in UK Biobank participants [30], which is in line with the proposal that brisk walkers are likely to be tall, thin and strong. Furthermore, *GDF5* rs143383-C (in high LD with *GDF5* rs143384-G) is a protective factor with respect to susceptibility to knee osteoarthritis [31] and stress fractures [33]. Thus, it might be speculated that rs143384-G might protect from a potential injury [34], possibly favoring the training flow of sprinters.

The TGS is a simple scoring model used to assess genetic predispositions for complex traits. Although it has some limitations (e.g., all SNPs were given the same weight in the total score), it represents an integrative association of multiple polymorphisms. Higher TGS values indicate a more favorable polygenic profile and suggest a partial genetic basis for the achievement of elite status in performance-relevant traits. Here, we identified that those with a higher TGS have increased odds of being an elite sprinter. As expected, the TGS computed using the 15 most relevant SNPs showed greater accuracy in identifying athletes (compared to the TGS based on all 70 SNPs, of which some SNPs showed an association in the opposite direction to the study hypothesis). It is well-established that the differences between groups become stronger when polymorphisms more relevant to the athlete group under analysis are accounted for [35]. We did not have an independent group to replicate our score findings, but it is expected that if the associations are similar in independent groups, the score will have a similar accuracy [36]. Nevertheless, it should be noted that both TGS models (computed using all 70 HSWAs or computed using only the 15 SNPs associated with brisk walking and sprint athlete status) were able to identify most sprinters when considering moderate-to-high scores.

The finding that the TGS computed using 70 SNPs also differentiates sprinters from controls was interesting and strengthens the relationship between HSWAs and sprinter athlete status, as the consideration of a large number of non-associated SNPs (in our sprinter cohort) did not eliminate its discriminatory power. Some potential candidate SNPs individually may not be associated with athletic status, but in interaction with other SNPs could have a main effect on the trait [36], as we have recently seen with testosterone-increasing alleles [37]. Furthermore, it cannot be ruled out that some non-associated SNPs in our study may have an association in independent and larger samples. Different environmental (through epigenetic mechanisms) and population (ethnic) aspects, for example, could result in associations with SNPs which our study was not sensitive enough to detect. Furthermore, it can be suggested that some of the non-associated SNPs in our study may not affect sprinting ability at all because of a lack of association with power-related phenotypes, such as muscle mass, strength, muscle fiber composition, testosterone levels, height and many others. It is noteworthy that there are several other polymorphisms in pathways that were not within the scope of this study that also contribute to the genetic architecture of elite power athletes [38].

Our study does have limitations. First, our sample size of athletes was relatively small (*n* = 137). This is quite a common problem with studies involving only elite athletes. Second, the control group consisted of people older than the athletes (43.1 ± 3.7 vs. 24.7 ± 3.7 years). Although age is a significant factor affecting allelic frequencies (of genes associated with longevity) in the population, one might expect this effect in older people (e.g., 60 years and older). Third, it has emerged that epigenetic factors (which have not been studied in the current work) regulating gene expression without changes in DNA sequences have an important role in the response to exercise training and the predisposition to compete at elite level [10]. Finally, as in all such studies, extension to and replication within other ethnic groups is proposed.

## 5. Conclusions

In this study, we have identified for the first time a shared genetic background between self-reported brisk walking pace and sprint athlete status. In an assessment of a cohort of elite sprinters, a total of 15 SNPs associated with self-reported brisk walking pace were found to be overrepresented in sprinters. Many of these SNPs have also been shown to be associated with other traits relevant to sprint performance in the UK Biobank, including increased height, fat-free mass and handgrip strength. Although replication studies are warranted, our study indicates that genetically determined walking pace may be a good predictor of sprinting ability. The results of this study potentially represent tentative steps towards our understanding of which genetic variants may predispose to enhanced sprint performance, suggesting that, if this information could be replicated in other cohorts, it may hold value in identifying talented future performers.

## Figures and Tables

**Table 1:** Fifteen SNPs related to self-reported walking pace associated with sprinter status.

			Consequence		HSWA (%)			*p*-Value (Allele Model)
Chr.	Gene	SNP	Type	HSWA	Sprinters	Controls	Diff.	
1	*ADAM15*	rs11264302	Intronic	G	55.8	42.9	0.129	0.003
1	*CCT3*	rs11548200	Exonic	T	95.6	89.7	0.058	0.009
1	*IGSF3*	rs699785	Intronic	A	30.7	15.5	0.152	0.00004
2	*FHL2*	rs55680124	ncRNA	C	88.3	82.9	0.053	0.078
3	*PPARG*	rs2920503	Intergenic	T	33.9	25.4	0.085	0.032
4	*SLC39A8*	rs13107325	Exonic	C	97.1	91.7	0.053	0.007
6	*C6orf106*	rs205262	Intronic	A	79.2	73.0	0.062	0.096
6	*E2F3*	rs4134943	Intronic	T	19.0	13.1	0.059	0.067
7	*AUTS2*	rs10452738	Intronic	A	75.5	68.3	0.072	0.063
10	*CRTAC1*	rs2439823	Intronic	A	54.0	40.9	0.131	0.003
14	*AKAP6*	rs12883788	Intergenic	C	56.9	48.4	0.085	0.050
14	*NRXN3*	rs8011870	ncRNA	G	67.2	59.9	0.073	0.085
19	*CRTC1*	rs11881338	Intronic	A	57.7	44.0	0.137	0.002
19	*ZNF568*	rs1667369	Intergenic	A	75.5	65.5	0.100	0.011
20	*GDF5*	rs143384	UTR5	G	51.1	38.9	0.122	0.005

Abbreviations: Chr. = Chromosome; Diff. = Difference between fractions (Sprinters-Controls); HSWA = High-speed-walking allele. The significance threshold for nominal associations was set at *p* < 0.05, while the significance threshold adjusted for multiple comparisons was set at *p* < 0.00018.

**Table 2:** Genotype distribution of SNPs related to self-reported walking pace associated with sprinter status.

		Alleles		Genotype Distribution (%)			*p*-Values		
Gene	SNP	1/2	Group	1/1	1/2	2/2	Cod.	Dom.	Rec.
*ADAM15*	rs11264302	A/G	Controls	35.7	42.9	21.4	0.005	0.001	0.116
			Sprinters	18.2	51.8	29.9	—	—	—
*CCT3*	rs11548200	T/C	Controls	81.7	15.9	2.4	0.045	0.014	0.274
			Sprinters	92.0	7.3	0.7	—	—	—
*IGSF3*	rs699785	G/A	Controls	71.4	26.2	2.4	0.0002	0.0001	0.026
			Sprinters	47.4	43.8	8.8	—	—	—
*FHL2*	rs55680124	C/T	Controls	70.6	24.6	4.8	0.032	0.269	0.010
			Sprinters	76.6	23.4	0.0	—	—	—
*PPARG*	rs2920503	C/T	Controls	55.6	38.1	6.3	0.098	0.095	0.065
			Sprinters	45.3	41.6	13.1	—	—	—
*SLC39A8*	rs13107325	C/T	Controls	84.9	13.5	1.6	0.033	0.014	0.139
			Sprinters	94.2	5.8	0.0	—	—	—
*C6orf106*	rs205262	A/G	Controls	56.3	33.3	10.3	0.098	0.348	0.032
			Sprinters	62.0	34.3	3.6	—	—	—
*E2F3*	rs4134943	C/T	Controls	73.8	26.2	0.0	0.032	0.238	0.010
			Sprinters	67.2	27.7	5.1	—	—	—
*AUTS2*	rs10452738	A/G	Controls	44.4	47.6	7.9	0.068	0.024	0.846
			Sprinters	58.4	34.3	7.3	—	—	—
*CRTAC1*	rs2439823	G/A	Controls	34.9	48.4	16.7	0.010	0.009	0.023
			Sprinters	20.4	51.1	28.5	—	—	—
*AKAP6*	rs12883788	C/T	Controls	25.4	46.0	28.6	0.121	0.230	0.048
			Sprinters	32.1	49.6	18.2	—	—	—
*NRXN3*	rs8011870	G/A	Controls	38.9	42.1	19.0	0.084	0.420	0.026
			Sprinters	43.8	46.7	9.5	—	—	—
*CRTC1*	rs11881338	T/A	Controls	31.7	48.4	19.8	0.009	0.011	0.012
			Sprinters	18.2	48.2	33.6	—	—	—
*ZNF568*	rs1667369	A/C	Controls	42.1	46.8	11.1	0.036	0.022	0.073
			Sprinters	56.7	38.7	5.1	—	—	—
*GDF5*	rs143384	A/G	Controls	38.1	46.0	15.9	0.015	0.006	0.073
			Sprinters	22.6	52.6	24.8	—	—	—

Abbreviations: Cod. = Codominant model; Dom. = Dominant model; Rec. = Recessive model. Underlined alleles indicate high-speed-walking alleles (HSWAs).

**Table 3:** The total genotype score (TGS) computed using all 70 SNPs related to self-reported walking pace or only the 15 SNPs associated with brisk walking and sprinter status.

	TGS (70 SNPs)		TGS (15 SNPs)	
	Sprinters	Controls	Sprinters	Controls
Number of participants	137	126	137	126
Minimum score (number of favorable alleles)	45.7 (64)	42.9 (60)	40.0 (12)	23.3 (7)
Maximum score (number of favorable alleles)	62.9 (88)	60.0 (84)	86.7 (26)	73.3 (22)
Mode (number of favorable alleles)	55.0 (77)	50.7 (71)	63.3 (19)	56.7 (17)
Mean (SD)	53.7 (3.4)	51.3 (3.8)	62.5 (7.4)	53.3 (9.1)
Difference between means; *p*-value	2.4 ± 0.4; *p* < 0.0001		9.2 ± 1.0; *p* < 0.0001	
Skewness (SE)	0.005 (0.207)	−0.095 (0.216)	−0.024 (0.207)	−0.289 (0.216)
Kurtosis (SE)	−0.391 (0.411)	−0.582 (0.428)	0.811 (0.411)	0.004 (0.428)
Area under the ROC curve (95% CI); *p*-value	0.673 (0.608–0.738); *p* < 0.0001		0.781 (0.725–0.837); *p* < 0.0001	

## Data Availability

The data presented in this study are available on request from the corresponding author.

## Supplementary Materials

The following supporting information can be downloaded at: https://www.mdpi.com/article/10.3390/genes13101710/s1, Table S1: Overview of SNPs and prior associations. Table S2: Association of top 15 SNPs with exercise and health-related traits. Table S3: Genes containing SNPs associated with sprint athlete status. Table S4: Association of top 15 SNPs with muscle fiber characteristics in 67 physically active men.
